# The Effects of p38 MAPK Inhibition Combined with G-CSF Administration on the Hematoimmune System in Mice with Irradiation Injury

**DOI:** 10.1371/journal.pone.0062921

**Published:** 2013-04-30

**Authors:** Deguan Li, Yueying Wang, Hongying Wu, Lu Lu, Xiaochun Wang, Junling Zhang, Heng Zhang, Saijun Fan, Feiyue Fan, Daohong Zhou, Aimin Meng

**Affiliations:** 1 Institute of Radiation Medicine, Chinese Academy of Medical Science and Peking Union Medical College, Tianjin Key Laboratory of Molecular Nuclear Medicine, Tianjin, China; 2 Division of Radiation Health, Department of Pharmaceutical Sciences and Winthrop P. Rockefeller Cancer Institute, University of Arkansas for Medical Sciences, Little Rock, Arkansas, United States of America; Emory University, United States of America

## Abstract

The acute and residual (or long-term) bone marrow (BM) injury induced by ionizing radiation (IR) is a major clinic concern for patients receiving conventional radiotherapy and victims accidentally exposed to a moderate-to-high dose of IR. In this study, we investigated the effects of the treatment with the p38 inhibitor SB203580 (SB) and/or granulocyte colony-stimulating factor (G-CSF) on the hematoimmune damage induced by IR in a mouse model. Specifically, C57BL/6 mice were exposed to a sublethal dose (6 Gy) of total body irradiation (TBI) and then treated with vehicle, G-CSF, SB, and G-CSF plus SB. G-CSF (1 µg/mouse) was administrated to mice by intraperitoneal (ip) injection twice a day for six successive days; SB (15 mg/kg) by ip injection every other day for 10 days. It was found that the treatment with SB and/or G-CSF significantly enhanced the recovery of various peripheral blood cell counts and the number of BM mononuclear cells 10 and 30 days after the mice were exposed to TBI compared with vehicle treatment. Moreover, SB and/or G-CSF treatment also increased the clonogenic function of BM hematopoietic progenitor cells (HPCs) and the frequency of BM lineage**^−^**Sca1^+^c-kit^+^ cells (LSK cells) and short-term and long term hematopoietic stem cells (HSCs) 30 days after TBI, in comparison with vehicle treated controls. However, the recovery of peripheral blood B cells and CD4^+^ and CD8^+^ T cells was not significantly affected by SB and/or G-CSF treatment. These results suggest that the treatment with SB and/or G-CSF can reduce IR-induced BM injury probably in part via promoting HSC and HPC regeneration.

## Introduction

Hematoimmune injury is one of the most important side effects of radiotherapy. Some patients receiving radiotherapy might develop both acute and long-term myelosuppression [1], [2]. Unfortunately, an effective treatment against ionizing radiation (IR)-induced bone marrow (BM) damage has yet to be developed [3], [4].

The p38 mitogen-activated protein kinase (p38) pathway can be activated in response to a variety of extracelluar stimuli, particularly to cellular stress such as osmotic shock, hypoxia, and IR [5], [6]. Studies have shown that p38 plays a critical role in regulating cell survival and regeneration following exposure to IR. In the hematopoietic system, the p38 pathway plays an essential role in regulation of erythropoiesis and myelopoiesis [7], [8], [9]. Moreover, p38 activation has been implicated in mediating BM suppression in various hematopoietic pathologic conditions, such as aplastic anemia (AA) and myelodysplastic syndromes (MDS). Inhibition of p38 either with a pharmacological inhibitor or by a genetic approach has been exploited for AA and MDS treatment because p38 inhibition can inhibit hematopoietic stem cell (HSC) apoptosis and stimulate hematopoietic progenitor cell (HPC) proliferation [10], [11]. In addition, p38 inhibition can rescue the defects of HSCs from ATM mutant and Foxo3 knockout mice [12], [13]. Our previous study has also shown that inhibition of p38 can promote *ex vivo* HSC expansion and attenuate hematopoietic cell senescence induced by IR [14]. Extensive studies have provided evidence that G-CSF is radioprotective in mice if it is administered before or shortly after exposure to IR [15]. In addition, some reports have shown that G-CSF can significantly increase HPC/HSC mobilization, stimulate granulopoiesis, and increase neutrophil antimicrobial activities [16], [17], [18], [19]. Exposure to a moderate or a high dose of total body irradiation (TBI) induces not only acute BM suppression but also residual (or long-term) BM injury. Our previous research has shown that the combined therapy with a p38 inhibitor and G-CSF could reduce TBI-induced lethality in part by mitigating TBI-induced acute BM injury [20]. However, the effects of p38 inhibition and/or G-CSF treatment on TBI-induced long-term BM suppression were unknown and thus, were investigated in the present studies.

## Materials and Methods

### Animals

Male C57BL/6 mice were purchased from Vital River (Beijing, China) and housed in the certified animal facility at the Institute of Radiation Medicine of the Chinese Academy of Medical Sciences (CAMS). All mice were used at approximately 8–12 weeks of age. All experimental procedures were performed with the approval of the Animal Use Committee at the Institute of Radiation Medicine of CAMS.

### Irradiation and SB and/or G-CSF Treatment

Mice were exposed to 6 Gy TBI from a ^137^Cs source housed in an Exposure Instrument Cammacell-40 (Atomic Energy of Canada Lim, Ottawa, Canada) at a dose-rate of 0.78 Gy per minute. After irradiation, animals were returned to the animal facility for daily observation.

SB 203580 (SB, LC Laboratories, Woburn, MA, USA), a specific p38 inhibitor, was dissolved in a saline solution containing 30% DMSO. G-CSF (JZJY Cor., Hangzhou, China) was diluted to 5 µg/ml with a saline solution containing 30% DMSO. For SB treatment, mice were given SB at 15 mg/kg via intraperitoneal injection (ip) 24 h after irradiation, and then thereafter every other day for a total of 5 injections. For G-CSF treatment, mice were administered with G-CSF at a dose of 1 µg/mouse by ip injection at 2 h and 6 h on day 1 after irradiation, and then twice per day for 5 days. For the combined therapies, mice were given both SB and G-CSF as described above. As a control, mice were irradiated and then treated with vehicle (saline with 30% DMSO) in a similar manner as that described for SB and/or G-CSF treatment [20].

### Analysis of Peripheral Blood Cell and BM Mononuclear Cell (BMMNC) Counts

Blood samples were obtained from the orbital sinus using a micro-pipette coated with the anticoagulant K_3_EDTA. BM cells were flushed from mouse femurs with Hank’s balanced salt solution (HBSS) after mice were euthanized by CO_2_ suffocation followed by cervical dislocation. The numbers of various blood cells and BMMNCs was counted using a pocH-100i hemocytometer (Sysmex, Kobe, Japan) and expressed as 10^9^/L and ×10^6^/femur, respectively.

### Organ Coefficient Calculation

The spleen and thymus were collected and weighted after mice were euthanized. The spleen and thymus coefficients were calculated by dividing individual organ weight (mg) with their body weight (g).

### Colony-forming Cells (CFC) Assay

The CFC assay was performed by culturing BM cells in MethoCult GF M3534 methylcellulose medium (StemCell Technologies, Vancouver, BC, Canada) according to the manufacturer’s instruction. The colonies of CFU-granulocyte macrophage (CFU-GM) with more than 30 cells were scored under an invert microscope after 7 days of culture. The results were expressed as the numbers of CFU-GM (×10^3^) per femur.

### Lymphocyte Phenotype Assay by Flow Cytometry

After red blood cells were lysed with BD Pharm Lyse™ Lysing Buffer (BD Biosciences, San Diego, CA, USA), the remaining cells in blood were washed twice with phosphate-buffered saline containing 1% bovine serum albumin (PBS-BSA) (pH 7.4) and re-suspended in 100 µl PBS-BSA solution. They were stained with phycoerythrin (PE)-conjugated anti-CD8, fluorescein isothiocyanate (FITC)-conjugated anti-CD4 antibodies, PE-cy5-conjugated anti-B220 (eBioscience Inc., San Diego, CA, USA) for 15 min at room temperature in the dark. The samples were analyzed by a Beckman Coulter flow cytometer (Beckman Coulter, Brea, CA, USA) using the Expo32 analysis software.

### Analysis of BM Lineage**^−^** Sca1^+^c-kit^+^ cells (LSK Cells) and HSCs by Flow Cytometry

BMMNCs were incubated with biotin-conjugated rat antibodies specific for murine CD5, Mac-1, CD45R/B220, Ter-119, and Gr-1 for 15 min at room temperature. After washed by PBS twice, the cells were stained with APC-Cy7-conjugated Streptavidin, FITC-conjugated anti-CD34, PE-conjugated anti-Flt3, PE-Cy7-conjugated anti-Sca1, and Alexa Fluor 700-conjugated anti-c-kit antibodies (eBioscience, San Diego, CA, USA) and analyzed by flow cytometry. The numbers of a particular cell population were calculated using the following formula: cell numbers/femur = BMMNCs/femur×percentage of positive cells in BMMNCs.

### Statistical Analysis

The results were expressed as the mean ± standard deviations (SD). The data were analyzed by Student’s *t*-test or one-way analysis of variance (ANOVA). *p* value of less than 0.05 was considered significant.

## Results

### Treatment with SB and/or G-CSF Facilitates Hematologic Recovery After TBI

Hematopoietic cells are highly sensitive to IR and their injury is the primary cause of death after exposure to a dose of TBI in the range of 4–10Gy [20]. To investigate the effects of SB and/or G-CSF treatment on IR-induced BM injury, we first analyzed the peripheral blood cell counts at various times after 6Gy TBI. As shown in Table 1, the animals treated with SB, G-CSF and a combination of both had higher levels of WBC counts than the vehicle-treated mice at 10d post TBI. However, the differences among the mice receiving SB and/or G-CSF treatment were not statistically significant. In addition, G-CSF alone or in combination with SB slightly increased the platelet (PLT) counts. As shown in Table 2, the WBC counts in the peripheral blood from animals treated with SB and/or G-CSF were significantly higher compared to those in the vehicle-treated group at 30d after 6Gy TBI (p<0.05). The mice treated with SB plus G-CSF also had higher levels of RBC counts, hemoglobin (HGB) and hematocrit (HCT) in their blood than those treated with vehicle, G-CSF, or SB. Furthermore, the peripheral blood PLT counts were significantly higher in mice treated with SB alone or in combination with G-CSF than those in mice treated without SB. These findings suggest that G-CSF can promote WBC production while SB is more effective in increasing PLT production after TBI. Therefore, a combined therapy of SB and G-CSF may be more advantageous than treatment with either agent in facilitating hematopoietic recovery after TBI.

**Table 1:** Peripheral blood cell counts at 10d after 6Gy TBI.

Mice (n = 5)	WBC(10^9^/l)	RBC(10^12^/l)	HGB(g/l)	HCT(%)	PLT(10^9^/l)
**Ctr**	5.9±0.9	9.7±0.3	13.6±0.6	49.7±2.0	1120±69
**V**	0.1±0.0^a^	6.8±0.7^a^	9.8±1.1^a^	32.9±3.4^a^	56±10^a^
**G-CSF**	0.4±0.1^a,b^	7.2±0.5^a^	10.8±0.8^a^	35.3±2.3^a^	121±21^a,b^
**SB**	0.2±0.0^a,b^	6.5±0.5^a,c^	9.2±1.2^a,c^	30.2±3.9^a,c^	55±11^a,c^
**SB+G-CSF**	0.3±0.1^a,b,d^	7.2±0.7^a,d^	10.7±1.3^a,d^	35.6±3.8^a,d^	133±34^a,b,d^

Mice were treated with ip injection of vehicle (V), SB 203580 (SB), G-CSF (CSF), or both (C+S) after exposure to 6Gy TBI as described in the Methods. A group of sham-irradiated control mice was included as a control (Ctr). Blood were collected after the mice were euthanized 10 days after 6Gy TBI to count various blood cells. The data are expressed as mean± SD. a, *p*<0.05, vs. Ctr; b, *p*<0.05, vs. V; c, *p*<0.05, vs. CSF; d, *p*<0.05, vs. SB.

**Table 2:** Peripheral blood cell counts at 30d after 6Gy TBI.

Mice (n = 8)	WBC(10^9^/l)	RBC(10^12^/l)	HGB(g/l)	HCT(%)	PLT(10^9^/l)
**Ctr**	8.2±1.5	10.0±0.4	14.1±0.6	50.4±2.1	1282±272
**V**	2.3±0.3^a^	8.1±0.5^a^	12.1±0.7^a^	41.9±3.3^a^	793±224
**G-CSF**	3.1±0.9^a^	8.8±0.7^a^	12.5±0.7	43.3±2.9^a^	703±91^a^
**SB**	3.9±1.4^a,b^	8.8±0.2^a,b^	12.2±0.7^a^	43.2±1.9^a^	1022±180^a,c^
**SB+G-CSF**	3.2±0.6^a,b^	9.7±0.8^b,c,d^	13.9±1.7^b,c,d^	48.6±4.8^b,c,d^	901±201^a,c,d^

Mice were treated with ip injection of vehicle (V), SB 203580 (SB), G-CSF (CSF), or both (C+S) after exposure to 6Gy TBI as described in the Methods. A group of sham-irradiated control mice was included as a control (Ctr). Blood were collected after the mice were euthanized 30 days after 6Gy TBI to count various blood cells. The data are expressed as mean± SD. a, *p*<0.05, vs. Ctr; b, *p*<0.05, vs. V; c, *p*<0.05, vs. CSF; d, *p*<0.05, vs. SB.

### Effects of SB and/or G-CSF Treatment on BMMNC Counts

**Figure 1:**
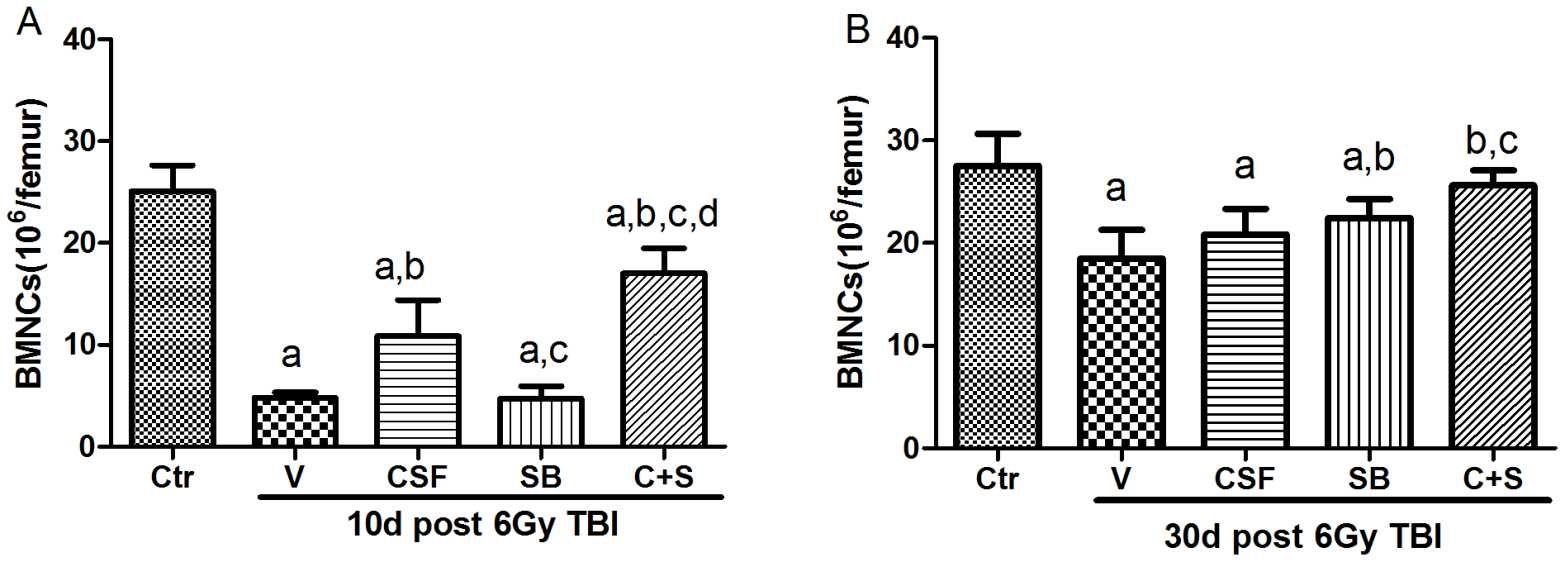
Effects of SB and/or G-CSF treatment on BMMNC counts. Mice were treated with ip injection of vehicle (V), SB 203580 (SB), G-CSF (CSF), or both (C+S) after exposure to 6Gy TBI as described in the Methods. A group of sham-irradiated control mice was included as a control (Ctr). BMMNCs were numerated after the mice were euthanized 10 days (A) and 30 days (B) after TBI. The data are expressed as mean± SD (n = 5). a, *p*<0.05, vs. Ctr; b, *p*<0.05, vs. V;c, *p*<0.05, vs. CSF; d, *p*<0.05, vs. SB.

To determine whether SB and/or G-CSF facilitate hematopoietic recovery after TBI via stimulating BM hematopoiesis, we analyzed BMMNC counts 10 and 30 days post 6Gy TBI. As demonstrated in Fig. 1A, treatment with SB and/or G-CSF significantly accelerated the recovery of BMMNC counts at 10d post 6Gy TBI. The numbers of BMMNCs in the mice treated with both SB and G-CSF were also higher than those in the SB or G-CSF treated mice. As shown in Fig. 1B, BMMNC counts in the irradiated mice receiving vehicle treatment were significantly lower than those in the un-irradiated control group at 30d post 6Gy TBI. Their numbers in irradiated mice treated with G-CSF alone were not significantly different from those in vehicle-treated mice after TBI. However, SB treatment either alone or in combination with G-CSF resulted in slightly higher levels of BMMNC counts than vehicle treatment. These results indicate that SB and G-CSF can be more effective in promoting hematopoiesis after 6Gy TBI than either agent.

### Effects of SB and/or G-CSF Treatment on BM CFU-GM

**Figure 2:**
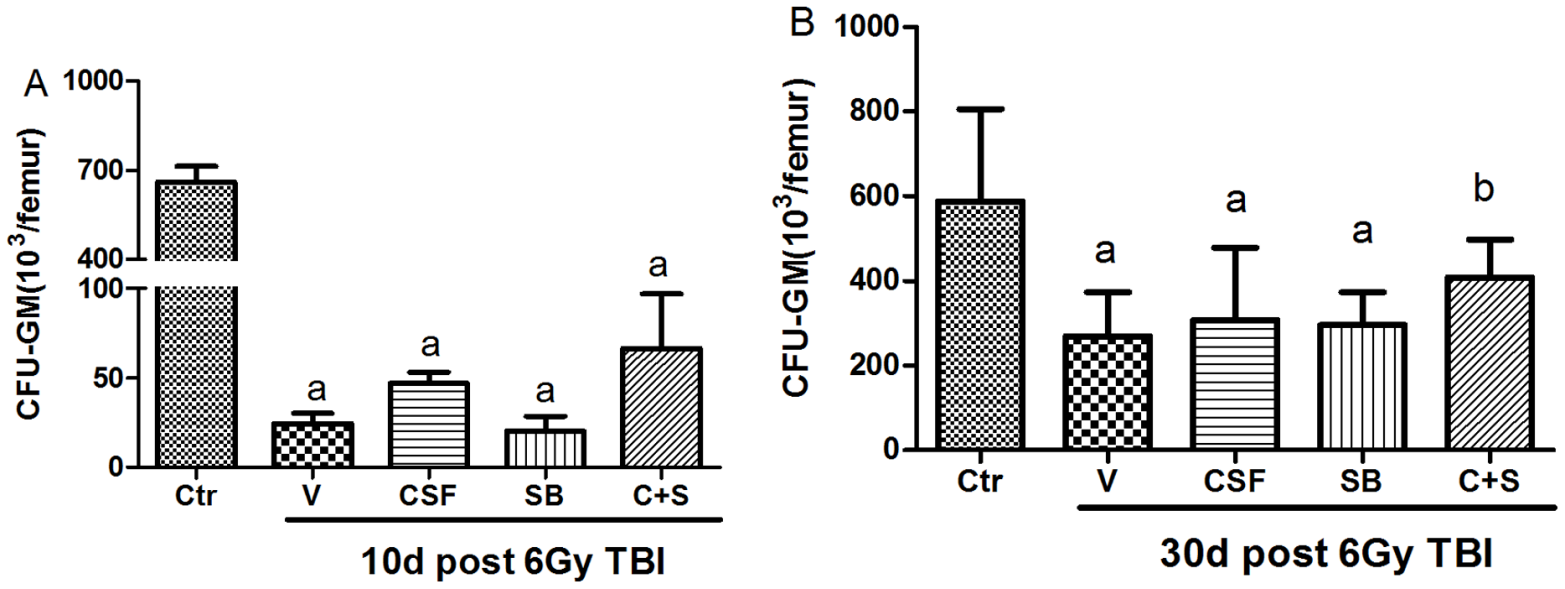
Effects of SB and/or G-CSF treatment on BM CFU-GM. Mice were treated with ip injection of vehicle (V), SB 203580 (SB), G-CSF (CSF), or both (C+S) after exposure to 6Gy TBI as described in the Methods. A group of sham-irradiated control mice was included as a control (Ctr). BMMNCs were collected from the mice after euthanization 10 days (A) and 30 days (B) after 6Gy TBI and cultured in MethoCult GF M3534 methylcellulose medium for analysis of CFU-GM. Results are expressed as the numbers of CFU-GM per femur and presented as mean± SD (n = 6). a, *p*<0.05, vs. Ctr; b, *p*<0.05, vs. V.

To determine whether SB and/or G-CSF can increase BM hematopoiesis after 6Gy TBI by stimulating HSC/HPC proliferation, we examined the effects of SB and/or G-CSF on BM CFU-GM. As shown in Fig. 2A, the frequencies of CFU-GM in BM cells from irradiated mice 10d post 6Gy TBI were significantly lower than those from control un-irradiated mice. The reduction in CFU-GM frequencies was slightly attenuated by the treatment with SB or G-CSF at 30d post 6Gy TBI as demonstrated in Fig. 2B. However, the effect of SB plus G-CSF on CFU-GM frequencies was significantly greater than that of either agent.

### Effects of SB and/or G-CSF Treatment on Immune Organ Coefficients

**Figure 3:**
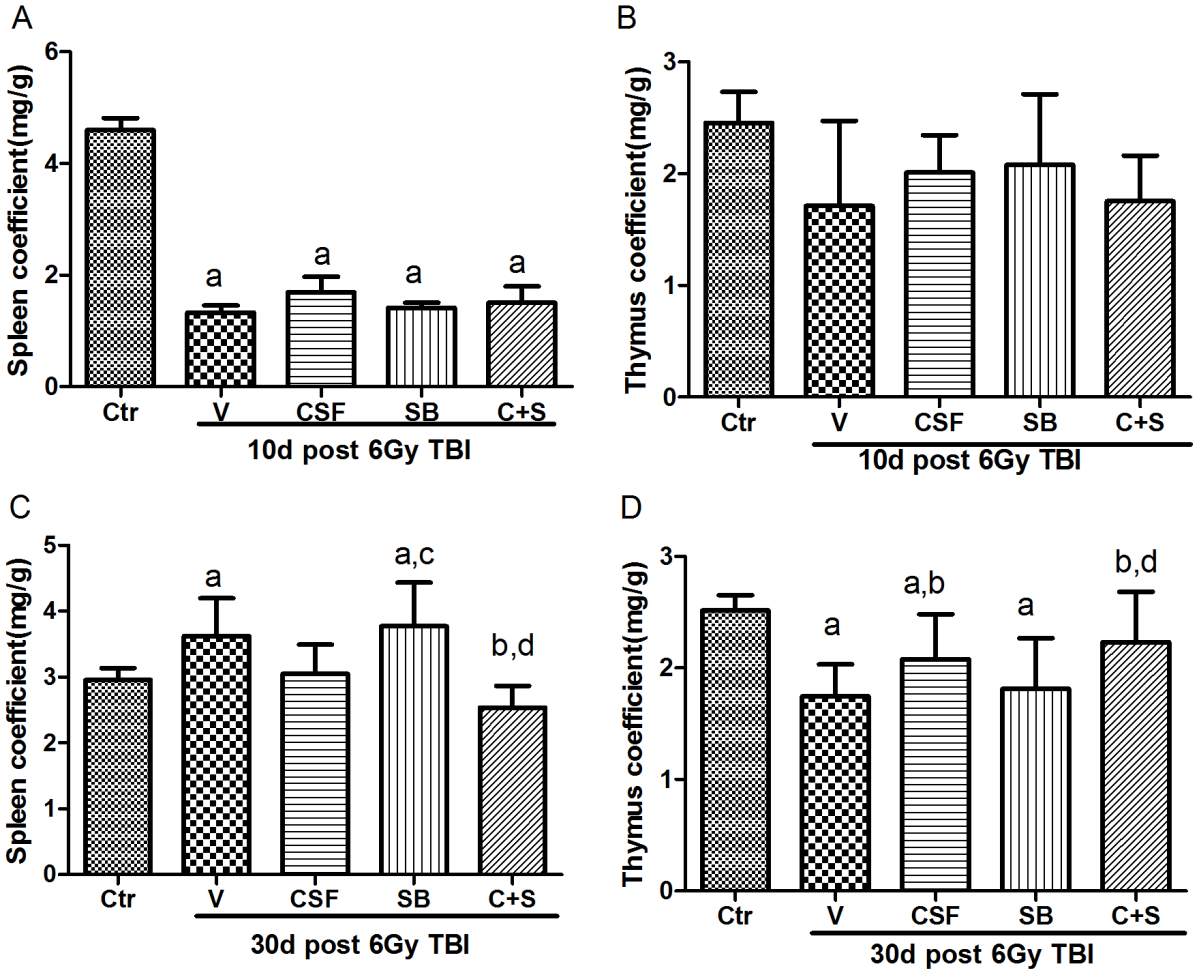
Effects of SB and/or G-CSF treatment on immune organ coefficients. Mice were treated with ip injection of vehicle (V), SB 203580 (SB), G-CSF (CSF), or both (C+S) after exposure to 6Gy TBI as described in the Methods. A group of sham-irradiated control mice was included as a control (Ctr). The spleen and thymus were collected and weighted after the mice were euthanized 10 days (A,B) and 30 days(C,D) after TBI. The organ coefficients were calculated by organ weight (mg)/mice weight (g). The data are expressed as mean± SD (n = 5). a, *p*<0.05, vs. Ctr; b, *p*<0.05, vs. V; c, *p*<0.05, vs. CSF; d, *p*<0.05, vs. SB.

To determine whether SB and/or G-CSF can promote immune cell recovery after 6Gy TBI, we first examined the effects of SB and/or G-CSF on immune organ coefficients but found no significant effects on the organ coefficients at 10d post 6Gy TBI (Fig. 3A and B). However, the spleen coefficients of irradiated mice receiving vehicle or SB treatment were slightly greater than those from all the other irradiated mice at 30d post 6Gy TBI (Fig. 3C). As shown in Fig. 3D, the thymus coefficients of irradiated mice receiving vehicle, G-CSF or SB treatment were significantly lower than those from control un-irradiated mice. The reduction was attenuated by the treatment with G-CSF alone and SB plus G-CSF compared to vehicle-treated mice 30 days after TBI.

### Effects of SB and/or G-CSF Treatment on Various Lymphocytes in Blood

**Figure 4:**
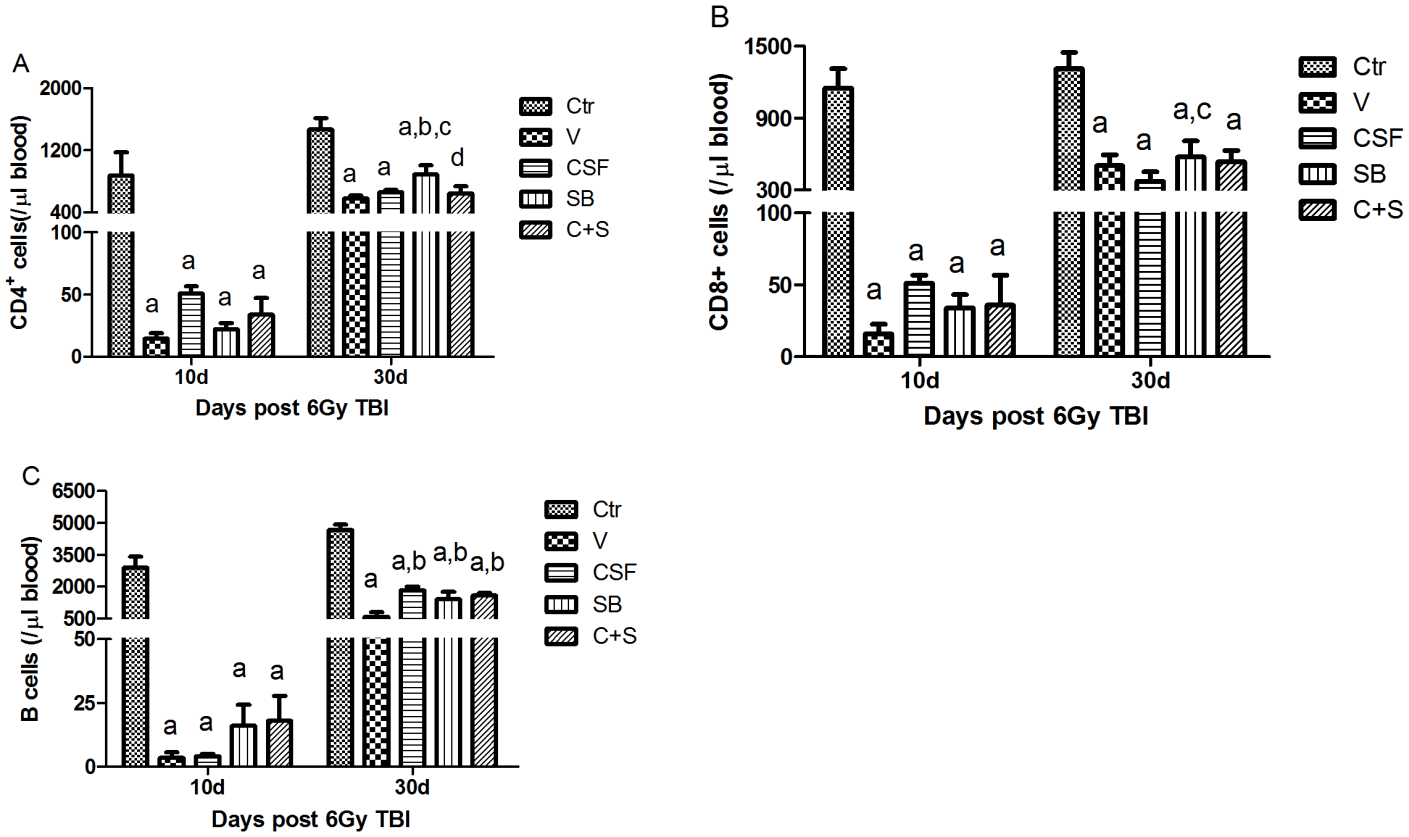
Effects of SB and/or G-CSF treatment on blood lymphocytes. Mice were treated with ip injection of vehicle (V), SB 203580 (SB), G-CSF (CSF), or both (C+S) after exposure to 6Gy TBI as described in the Methods. A group of sham-irradiated control mice was included as a control (Ctr). Blood was collected after the mice were euthanized 10 days and 30 days after 6Gy TBI and analyzed for CD4^+^ T cells, CD8^+^ T cells, and B cells by flow cytometry. The data are expressed as mean± SD (n = 5). A. CD4^+^ T cells; B. CD8^+^ T cells; and C. B cells. a, *p*<0.05, vs. Ctr; b, *p*<0.05, vs. V; c, *p*<0.05, vs. CSF; d, *p*<0.05, vs. SB.

Next, we examined the effects of SB and/or G-CSF on CD4^+^ T cell, CD8^+^ T cell, and B cell counts in blood. As shown in Fig. 4A, the numbers of CD4^+^ T cells in peripheral blood from irradiated mice received SB treatment were slightly higher than those from vehicle and G-CSF treated mice 30 days after TBI. As shown in Fig. 4B, the numbers of CD8^+^ cells in peripheral blood from irradiated mice receiving SB treatment were significantly higher than those from vehicle treated mice. As shown in Fig. 4C, the numbers of B cells in peripheral blood from irradiated mice receiving SB and/or G-CSF treatment were also slightly higher than those from vehicle treated mice 30 d post TBI. However, no significant difference in all these immune cells was found 10 days after TBI.

### Effects of SB and/or G-CSF Treatment on BM LSK Cells and HSCs

**Figure 5:**
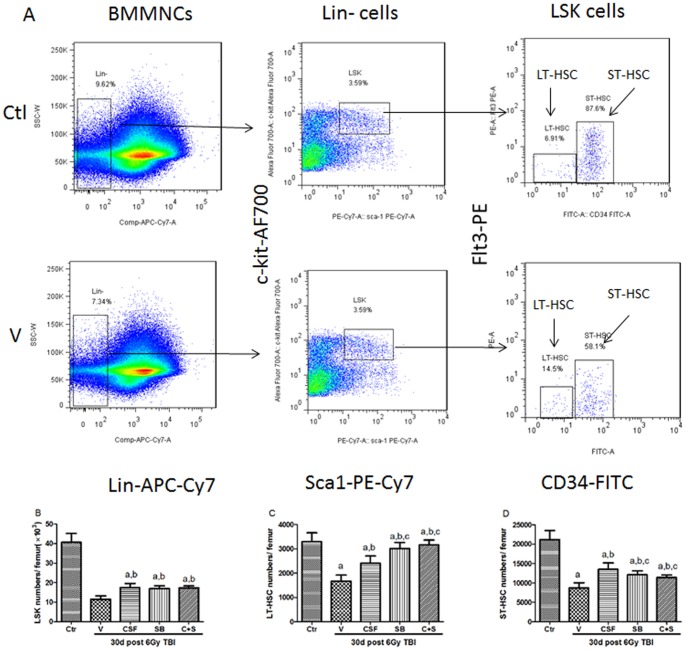
Effects of SB and/or G-CSF treatment on BM HSCs. Mice were treated with ip injection of vehicle (V), SB 203580 (SB), G-CSF (CSF), or both (C+S) after exposure to 6Gy TBI as described in the Methods. A group of sham-irradiated control mice was included as a control (Ctr). The BM cells were collected after the mice were euthanized 10 days and 30 days after 6Gy TBI and analyzed for LSK cells and HSCs by flow cytometry. The cell numbers were calculated using the following formula: cell numbers/femur = BMMNCs/femur×percentage of positive cells. (A) A representative gating strategy for flow cytometric analysis of LSK cells and HSCs; (B) numbers of LSK cells; (C) numbers of LT-HSCs (CD34**^−^**LSK cells); and (D) numbers of ST-HSCs (CD34^+^LSK cells). The data are expressed as mean± SD (n = 5). a, *p*<0.05, vs. Ctr; b, *p*<0.05, vs. V; c, *p*<0.05, vs. CSF; d, *p*<0.05, vs. SB.

To determine the effects of SB and/or G-CSF on HSCs, we analyzed the frequencies of lin**^−^**Sca-1^+^c-kit^+^ cells (LSK cells), long-term hematopoietic stem cells (LT-HSC, CD34**^−^**LSK cells), short term hematopoietic stem cells (ST-HSC, CD34^+^LSK cells) in BM 30 days after 6Gy TBI (Fig. 5A). The frequencies of BM LSK cells that are enriched for multipotent progenitor cells and HSCs, were higher in SB plus G-CSF treated mice than those in irradiated mice receiving vehicle treatment (Fig. 5B) [21]. Further analysis of the frequencies of LT-HSCs and ST-HSCs revealed that SB and/or G-CSF treatment promoted the recovery of these cells in BM after TBI in comparison with vehicle treatment (Fig. 5C and D). Compared to the G-CSF treated mice, the frequencies of LT-HSCs in SB and SB plus G-CSF treated mice were significantly greater than those in G-CSF treated mice.

## Discussion

Radiotherapy is the conventional approach in cancer treatment. Exposure to high doses of IR can cause long-term hematopoietic injury in part via induction of HSC senescence. In this study, we examined the effects of p38 inhibition with SB in combination with G-CSF on IR-induced acute and long-term hematopoietic injury. This is because it has been shown that treatment with G-CSF can ameliorate neutropenia induced not only by chemotherapy but also by radiotherapy [17], [22], [23]. In addition, activation of p38 also plays an important role in BM suppression under various pathological conditions including exposure to IR [24], [25], [26].

In a preliminary study, we found that IR and G-CSF can activate p38 in BM Lin- cells enriched with HSCs and HPCs and the activation can be abrogated by incubation of the cells with SB, a specific p38 inhibitor (Fig.S1). Therefore in the present study, we examined the effects of p38 inhibition with SB and/or G-CSF on IR-induced acute and long-term BM suppression in mice after exposure to a sublethal dose of TBI. Our results showed that there were some additive or synergistic effects of SB and G-CSF on hematopoietic and immune cell recovery in mice after they were exposed to a sublethal dose of TBI. It was found that treatment of the irradiated mice with G-CSF alone promoted the recovery of BMMNC, WBC and PLT counts at 10d post TBI, whereas the effects of G-CSF on these cells were not significant at 30d post TBI. These findings suggest that G-CSF treatment might be important in promoting the recovery of hematopoiesis immediately after TBI, which is consistent with the previous reports [27], [28], [29], [30]. It has been shown that p38 inhibition can modulate hematopoiesis as well [31]. In this study, we found that inhibition of p38 with SB alone promoted the recovery of WBC, RBC and PLT at 30d post irradiation, but its effects were not significant at 10d post irradiation. This finding suggests that that SB might be more helpful in promoting hematopoietic recovery at a later time after TBI. Therefore, a combination of SB and G-CSF appears slightly more effective in promoting both early as well as later hematopoietic recovery after TBI than either agent.

IR can also cause serious damage to the immune system. In our study, the immune organ coefficients, a commonly used indicator of immune suppression, in IR mice were significantly lower than those in control mice at 10d and 30d post TBI. However, the coefficients were restored by SB and G-CSF treatment to a level similar to that seen in unirradiated mice at day 30 post 6Gy TBI. A similar effect of SB and G-CSF treatment on CD4 T cell, CD8 T cell and B cell recovery was also observed in irradiated mice (Fig.S2). These results suggest that SB combined with G-CSF might also promote the recovery of immune injury induced by TBI.

The hematopoietic and immune stimulating effect of SB and/or G-CSF might be at the levels of HSCs and HPCs, because SB and/or G-CSF treatment increased the frequencies of BM LSK cells, ST-HSCs and LT-HSCs after TBI. This may be attributable to p38 inhibition mediated suppression of IR-induced HSC senescence as shown before [32], [33], [34]. In addition, p38 inhibition with SB combined with G-CSF also increased the proliferation of HPCs according to the results from the CFU assays. Our previous study also demonstrated that the combination treatment with SB and G-CSF could elevate the hematopoietic function of HSCs [20]. These observations suggested that SB combined with G-CSF not only promotes the recovery of HSCs and HPCs but also enhances the ability of HPCs to generate progeny. Therefore, G-CSF plus p38 inhibition has the potential to be exploited for mitigating IR-induced acute and long-term hematopoietic and immune injury.

## Supporting Information

Figure S1 **Effects of SB** **and/or G-CSF on p38 activation in lineage negative BM hematopoietic cells (Lin^−^ cells).** BMMNCs from normal C57BL/6 mice were incubated with biotin-conjugated rat antibodies specific for murine CD5, Mac-1, CD45R/B220, Ter-119, and Gr-1 (eBioscience Inc., San Diego, CA, USA). The labeled mature lymphoid and myeloid cells were depleted twice by incubation the cells with goat anti-rat IgG paramagnetic beads (Dynal Inc., Lake Success, NY, USA) at a bead:cell ratio of 4∶1 and were removed with a magnetic field. Lin**^−^** cells (2–3×10^6^/ml in RPMI1640 medium contained 10% FBS) were incubated with vehicle, SB (5 µM), G-CSF (10 ng/ml), or both at 37°C, 5% CO_2_, and 100% humidity for 30 min prior to exposure to 4 Gy IR *in vitro*. Two h after IR, the cells were fixed and permeablized with BD CytoFix/Cytoperm solutions (BD Biosciences, San Diego, CA, USA), and then stained with a rabbit anti-phosphorylated p38 (p-p38) antibody (Cell Signaling, Beverly, MA, USA) according to the manufacturers’ instructions. p-p38 staining was detected by a flow cytometer after staining with FITC–conjugated goat anti-rabbit IgG (Santa Cruz, Santa Cruz, CA, USA). (A-E) Representative flow cytometric analyses of p-p38 in Lin**^−^** cells with different treatments; (F) The merged flow cytometric analysis graph of A-E; and (G) The percentages of p-p38 positive cells under various treatment conditions are presented as mean± SD (n = 3). a, *p*<0.05, vs. Ctr; b, *p*<0.05, vs. V; c, *p*<0.05, vs. CSF; d, *p*<0.05, vs. SB.
(TIF)

Figure S2 **Effects of SB and/or G-CSF treatment on various lymphocytes in blood.** Mice were treated with ip injection of vehicle (V), SB 203580 (SB), G-CSF (CSF), or both (C+S) after exposure to 6Gy TBI as described in the Methods. A group of sham-irradiated control mice was included as a control (Ctr). Blood were collected after the mice were euthanized 30 days after 6Gy TBI and analyzed by flow cytometry. The data are expressed as mean± SEM (n = 5) of percentage of CD4^+^ T cells (A), CD8^+^ T cells (B), and B cells (C). a, *p*<0.05, vs. Ctr; b, *p*<0.05, vs. V; c, *p*<0.05, vs. CSF; d, *p*<0.05, vs. SB.
(TIF)
