# Supplementary figures and images for: The Effects of p38 MAPK Inhibition Combined with G-CSF Administration on the Hematoimmune System in Mice with Irradiation Injury
Source: PLoS One. 2013 Apr 30;8(4):e62921. doi: 10.1371/journal.pone.0062921 (PMC3639947; doi:10.1371/journal.pone.0062921)

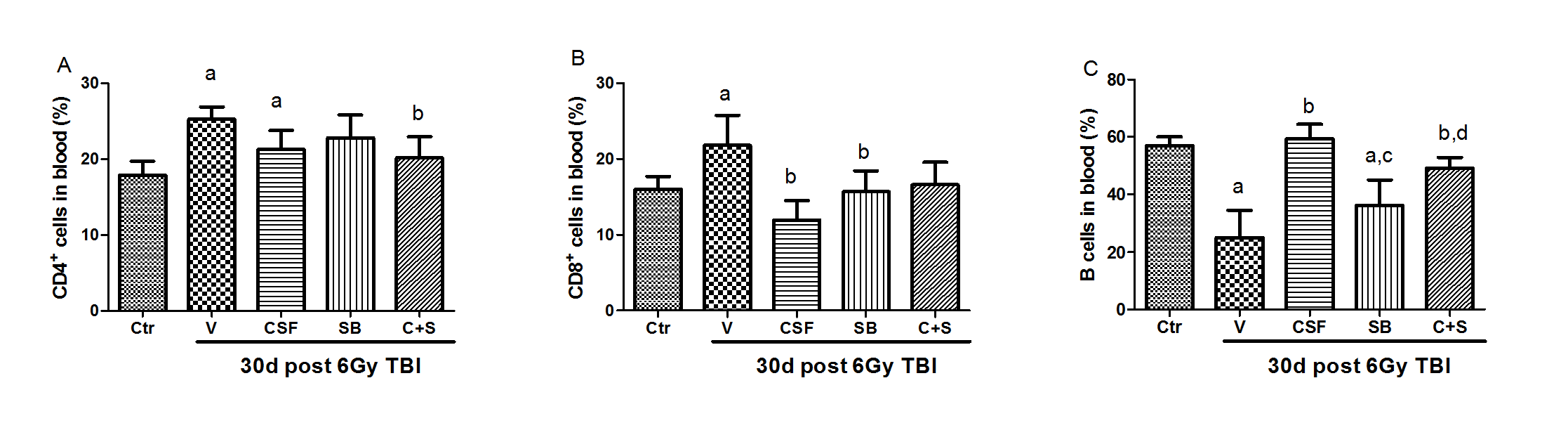

Supplement: Figure S1 — Effects of SB and/or G-CSF on p38 activation in lineage negative BM hematopoietic cells (Lin− cells). BMMNCs from normal C57BL/6 mice were incubated with biotin-conjugated rat antibodies specific for murine CD5, Mac-1, CD45R/B220, Ter-119, and Gr-1 (eBioscience Inc., San Diego, CA, USA). The labeled mature lymphoid and myeloid cells were depleted twice by incubation the cells with goat anti-rat IgG paramagnetic beads (Dynal Inc., Lake Success, NY, USA) at a bead:cell ratio of 4∶1 and were removed with a magnetic field. Lin− cells (2–3×10^6^/ml in RPMI1640 medium contained 10% FBS) were incubated with vehicle, SB (5 µM), G-CSF (10 ng/ml), or both at 37°C, 5% CO2, and 100% humidity for 30 min prior to exposure to 4 Gy IR in vitro. Two h after IR, the cells were fixed and permeablized with BD CytoFix/Cytoperm solutions (BD Biosciences, San Diego, CA, USA), and then stained with a rabbit anti-phosphorylated p38 (p-p38) antibody (Cell Signaling, Beverly, MA, USA) according to the manufacturers’ instructions. p-p38 staining was detected by a flow cytometer after staining with FITC–conjugated goat anti-rabbit IgG (Santa Cruz, Santa Cruz, CA, USA). (A-E) Representative flow cytometric analyses of p-p38 in Lin− cells with different treatments; (F) The merged flow cytometric analysis graph of A-E; and (G) The percentages of p-p38 positive cells under various treatment conditions are presented as mean± SD (n = 3). a, p<0.05, vs. Ctr; b, p<0.05, vs. V; c, p<0.05, vs. CSF; d, p<0.05, vs. SB. (TIF) [file pone.0062921.s001.tif]

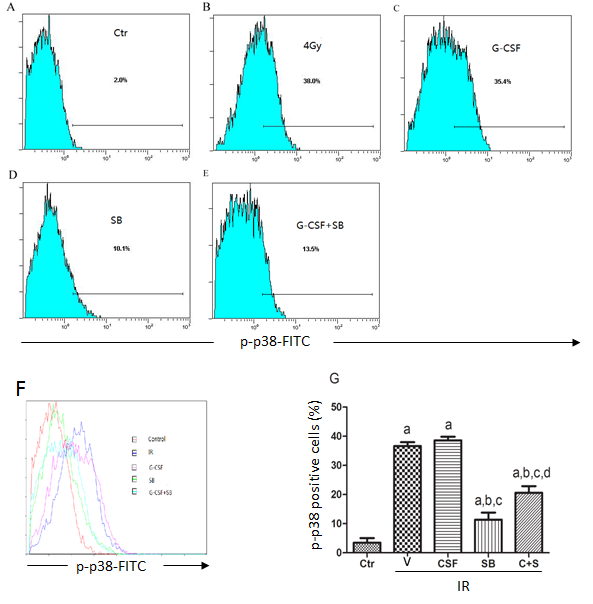

Supplement: Figure S2 — Effects of SB and/or G-CSF treatment on various lymphocytes in blood. Mice were treated with ip injection of vehicle (V), SB 203580 (SB), G-CSF (CSF), or both (C+S) after exposure to 6Gy TBI as described in the Methods. A group of sham-irradiated control mice was included as a control (Ctr). Blood were collected after the mice were euthanized 30 days after 6Gy TBI and analyzed by flow cytometry. The data are expressed as mean± SEM (n = 5) of percentage of CD4+ T cells (A), CD8+ T cells (B), and B cells (C). a, p<0.05, vs. Ctr; b, p<0.05, vs. V; c, p<0.05, vs. CSF; d, p<0.05, vs. SB. (TIF) [file pone.0062921.s002.tif]
